# Spinal Anesthesia for Knee Arthroscopy Using Isobaric Bupivacaine and Levobupivacaine: Anesthetic and Neuroophthalmological Assessment

**DOI:** 10.1155/2014/349034

**Published:** 2014-02-20

**Authors:** Monica del-Rio-Vellosillo, Jose Javier Garcia-Medina, Antonio Abengochea-Cotaina, Maria Dolores Pinazo-Duran, Manuel Barbera-Alacreu

**Affiliations:** ^1^Department of Anesthesia, University Hospital Virgen de la Arrixaca, Ctra. Madrid-Cartagena, s/n, El Palmar, 30120 Murcia, Spain; ^2^Department of Ophthalmology and Optometry, School of Medicine, University of Murcia, Avenida Intendente Jorge Palacios 1, 30003 Murcia, Spain; ^3^Department of Ophthalmology, General University Hospital Reina Sofia, Avenida Intendente Jorge Palacios 1, 30003 Murcia, Spain; ^4^Department of Anesthesia, University Hospital La Fe, Bulevar del Sur, 46026 Valencia, Spain; ^5^Department of Ophthalmology, University School of Medicine, University of Valencia, Avenida Blasco Ibáñez 15-17, 46010 Valencia, Spain

## Abstract

*Introduction.* The aim of the study was to compare the sensory, motor, and neuroophthalmological effects of isobaric levobupivacaine and bupivacaine when intrathecally administered. *Materials and Methods.* A prospective, double-blind, randomized study with 60 ASA grade I-II patients aged 18–65 years awaiting knee arthroscopy under spinal anesthesia. Patients received 12.5 mg of isobaric bupivacaine or levobupivacaine. Several features were recorded. *Results.* No significant intergroup differences were observed for ASA classification, time to micturate, demographic data, surgery duration, and patient/surgeon satisfaction. Similar hemodynamic parameters and sensory/motor blockade duration were found for both groups. There were no neuroophthalmological effects in either group. Sensory (*P* = 0.018) and motor blockade onset (*P* = 0.003) was faster in the bupivacaine group. T6 (T2–T12) and T3 (T2–T12) were the highest sensory block levels for the levobupivacaine and bupivacaine groups, respectively (*P* = 0.008). It took less time to regain maximum motor blockade in the bupivacaine group (*P* = 0.014), and the levobupivacaine group required use of analgesia earlier (*P* = 0.025). *Conclusions.* Isobaric bupivacaine and levobupivacaine are analogous and well-tolerated anesthetics for knee arthroscopy. However, for bupivacaine, sensory and motor blockade onset was faster, and greater sensory blockade with a longer postoperative painless period was achieved.

## 1. Introduction

Bupivacaine is considered the gold standard long-acting local anesthesia for most locoregional procedures. Levobupivacaine is an amide local anesthetic agent. As a pure S-enantiomer of racemic bupivacaine, it is the most recent long-acting local anesthetic agent to have been introduced for clinical use. Levobupivacaine is an attractive alternative to bupivacaine because its toxicity for the cardiovascular and central nervous systems (CNS) is lower [1–6].

Both anesthetics share many pharmacokinetic properties. Therefore, preliminary clinical experience reveals that the efficacy of both local anesthetics is more or less equal [7–13]. Volunteers were recruited to assess the clinical profile of spinal bupivacaine and levobupivacaine. Several published studies have compared solutions of 0.5% isobaric bupivacaine and levobupivacaine without an adjunct in spinal anesthesia via different surgical techniques: hip/knee replacement [7, 8], urological [9–11], lower abdominal, and lower extremity surgery [12].

Knowledge of the anatomy and vertebral column content, particularly the cerebrospinal system, is vital given the neuroophthalmological complications that may occur after spinal anesthesia. Batson's studies demonstrate how the cerebrospinal system continues from the pelvis and the dorsal spinal to the brain: it consists in the vertebral venous system or Batson's plexus, and the intracranial venous system and also includes the ophthalmological veins supplying the optic nerve (II cranial nerve) and oculomotor cranial nerves (III, IV, and VI cranial nerves) [14]. The cerebrospinal system has no valves, so flow is bidirectional. Cases of neuroophthalmological side effects with spinal anesthesia have been described [15–23].

The aim of this randomized, double-blind, prospective study was to provide further observations of these local anesthetic agents by comparing the clinical and anesthetic properties of levobupivacaine and bupivacaine and by assessing neuroophthalmological effects in spinal anesthesia for knee arthroscopy in one study. As far as we are aware, this is the first time that a clinical prospective study compares isobaric levobupivacaine and bupivacaine in knee arthroscopy and contemplates the perioperative neuroophthalmological effects of both drugs.

## 2. Materials and Methods

Sixty ASA I-II (the American Society of Anesthesiologists scale) patients aged 18–65 years, awaiting knee arthroscopy with spinal anesthesia, were enrolled in this prospective, randomized, double-blind study. The exclusion criteria were contraindication to spinal anesthesia, hypersensitivity to amide local anesthetic, a history of alcohol or drug abuse, uncontrolled hypertension, neurological, musculoskeletal, and ocular diseases, morbid obesity, and difficulties in understanding the study protocol. Approval was obtained from the institutional review board of the University Hospital La Fe, Valencia, Spain. All the participants signed a written informed consent.

Patients were randomly allocated to two groups, one receiving 2.5 mL of isobaric bupivacaine (Svedocain: bupivacaine hydrochloride, Inibsa laboratorios, Spain) 5 mg/mL (Group B) and the other was administered 2.5 mL isobaric levobupivacaine (Chirocaine: levobupivacaine hydrochloride, Abbot Laboratories, UK) 5 mg/mL (Group L) for spinal anesthesia in accordance with a computer-generated randomization list sealed in envelopes. The drugs were approved for neuraxial administration by the European Medicines Agency (EMA).

An anesthesiologist, who is not involved in patient care, prepared the anesthetic solution immediately prior to injection.

When patients arrived at the anesthetic room, complete ocular examination was performed, including the tests that follow, and in this order [24]: (1) near best-corrected visual acuity of each eye measured on a decimal scale; Rosenbaum test; (2) central vision of each eye assessment; Amsler grid test; (3) extrinsic ocular motility assessment plus examination of the nine diagnostic positions of gaze; (4) Bielschowsky test; (5) intrinsic ocular motility of each eye with flashlight by considering direct and consensual pupillary reflexes.

Then an 18-gauge intravenous cannula with 7 mL/kg of lactated Ringer solution and 0.03 mg/kg of midazolam was inserted before spinal anesthesia. Patients were intraoperatively administered with 10 mL/kg/h of lactated Ringer solution.

Both local anesthetics were administered intrathecally by a 29-gauge Quincke needle (BD Medical system, FrankinLakes, NJ, USA) at the L3-L4 interspace with patients in a sitting position. The distal needle port was oriented cranially and a median puncture was performed under aseptic conditions.

Intrathecal injections were carried out slowly for 15 seconds without barbotage or aspiration. Immediately afterward, patients were turned to a supine position and a pillow was placed beneath their heads.

The monitored parameters included noninvasive arterial pressure, heart rate, electrocardiogram, and pulse oximetry, before the spinal anesthesia conduct, then every 3 minutes for 15 minutes, and thereafter every 5 minutes until the procedure ended. Loss of sensation to cold spray (ethyl chloride) was employed to monitor sensory blockade. An amended Bromage scale (MBS 0 = no paralysis, able to flex hips/knees/ankles; 1 = able to move knees, unable to raise extended legs; 2 = able to flex ankles, unable to flex knees; 3 = unable to move any part of the lower limb) was utilized to assess motor blockade. Sensory and motor blockades were done every 1 min after spinal anesthesia until the greater sensory and motor blockade was achieved and then postoperatively every 30 min until the sensory and motor variables had returned to normal.

The sensory blockade onset time was assessed by referring to the interval between puncture and sensory blockade T12. The times to achieve the sensory blockades of T8 and T4 were recorded. The motor blockade onset time was assessed by referring to the interval between puncture and Bromage = 1. The maximum motor blockade degree and the highest sensory blockade level were recorded. The time from drug administration until MBS returned to zero, or 2-segment sensory regression, was defined as motor or sensory blockade duration.

All the interventions took place after helping patients into a supine position and by ensuring that the leg to be intervened was hanging down and had a tourniquet inflated to 350 mmHg.

Another complete ocular examination and the aforementioned assessments were performed in each patient 5 minutes after initiating spinal anesthesia.

The surgical procedure commenced 20 min after spinal anesthesia. Addition of any sedation drugs was recorded. The protocol was changed to general anesthesia if the blockade failed or proved inappropriate.

When blood pressure went below the baseline by more than 25%, it was treated as hypotension with incremental doses of 5 mg ephedrine I.V. and intravenous lactate Ringer's solution. If the heart rate was <45 bpm, it was treated as bradycardia with 0.5 mg atropine I.V. If SpO_2_ lowered to ≤92%, it was treated as hypoxemia with supplement oxygen via a face mask.

At the end of surgery, each patient completed a neuroophthalmological symptoms questionnaire (Table 1). It was completed by telephone at 24 h, 72 h, and 1 week after spinal anesthesia, when patients were also asked about any other adverse sequelae.

The scale to evaluate patient satisfaction was as follows: excellent (no intraoperatory pain at all), good (slight intraoperatory discomfort with no need for analgesia), fair (pain that required further analgesia with nonsteroidal anti-inflammatory drugs—NSAIDs), and poor (pain that required NSAIDs and opioids). Surgeons were also asked to estimate the operating conditions (degree of relaxing the lower member to be operated) on the following scale: excellent, good, fair, and poor.

Time to micturate was recorded in all the subjects. Postoperative pain was recorded every 30 minutes using a visual analogical scale (VAS) ranging from 0 (no pain at all) to 10 (maximal pain). If the pain score at rest was ≥4, dexketoprofen 50 mg/8 h I.V. and paracetamol 1 g/6 h I.V. were administered.

Sample size was determined as being consistent with previous similar studies so as to maintain the overall alpha error at <0.05 and statistical power of at least 80% [9, 11, 25–27]. SPSS, version 17.0, was used to statistically analyze the results obtained. A Kolmogorov-Smirnov test was done to assess all the variables for normality. The normally distributed data and nonnormally distributed data were expressed as means (SD) and medians (range), respectively. Mann-Whitney *U* tests (nonparametric data) and Student's *t*-tests (parametric data) were employed for the statistical analyses. ANOVA was used to compare more than two parametric variables, with Bonferroni correction for multiple comparisons. Fisher's exact or Chi-squared tests were utilized to make categorical comparisons. Ordinal data were compared using Sommer's “*d*” test. Survival curves were created with a Kaplan-Meier analysis and comparisons were made between these curves using the Breslow test. A *P* < 0.05 was taken as the a priori level of significance.

## 3. Results

Sixty patients were recruited (Group L, *n* = 31; Group B, *n* = 29). One patient in Group B was excluded due to technical failure in the blockade. The statistical analysis was therefore calculated with the remaining 59 patients (31 in Group B; 28 in Group L).

No significant differences between Groups L and B were found for type of surgery, ASA classification, or demographic data (Table 2).

**Figure 1:**
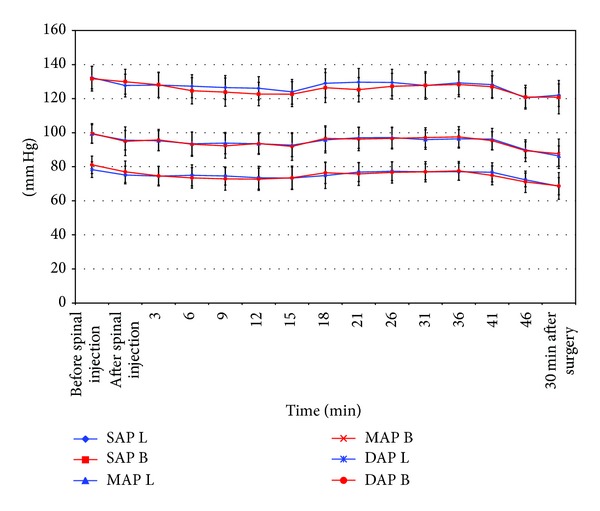
Comparison of hemodynamic effects before and up to 46 minutes after spinal anesthesia. Systolic arterial pressure (SAP), mean arterial pressure (MAP), and diastolic arterial pressure (DAP); bupivacaine (B); levobupivacaine (L).

Both groups presented analogous hemodynamic parameters before and during surgery, but there were no statistically significant differences (Figure 1). Once again, no statistical differences were observed for either group for the times (in min; median (range)) to achieve T8 (bupivacaine 5.5 (2–42), levobupivacaine 9 (1–25)), T4 (bupivacaine 4.5 (3–26), levobupivacaine 6 (4–53)), and for maximum sensory blockade (bupivacaine 14 (5–42), levobupivacaine 15 (2–53)). However, statistically significant differences were found between both groups for the time (in min; median (range)) to achieve T12 (bupivacaine 1.5 (1–10), levobupivacaine 3 (1-2)) and for the maximum upper sensory blockade level (bupivacaine T3 (T2–T12), levobupivacaine T6 (T2–T12)), but none were seen in the sensory regression pattern between both local anesthetics (Table 3).

For motor blockade, statistically significant differences were observed between both groups for the motor onset time (in min; median (range)) (bupivacaine 3 (1–15), levobupivacaine 7 (1–15)) and the maximum motor blockade time (in min; median (range)) (bupivacaine 15 (3–38), levobupivacaine 40 (6–83)). However, none were observed for the maximum and motor blockade regression patterns (Table 3).

No neuroophthalmological side effects were noted in either group before and after spinal anesthesia. Near best-corrected visual acuities values were the same before and after spinal anesthesia in all the patients in both Groups B and L (Table 4). Neither scotoma nor metamorphopsia was detected in any patient during the Amsler grid test. Additionally, no limitations in any eye in the nine diagnostic gaze positions were found and the Bielschowsky tests were normal in all cases. We documented no case of binocular diplopia. Finally, all the patients showed isochoric and normoreactive pupils for the pre- and postintrathecal anesthesia in both groups. Anesthesia was adequate and no patients required midazolam sedation during the intraoperative period. There was a similar interval between spinal injection and first voiding for both groups.

Statistically significant differences were found between both groups for the time (in min; median (range)) to require analgesic drugs after spinal anesthesia (bupivacaine 297 (146–444) levobupivacaine 247 (184–436)).

Surgeon satisfaction was 92.9% excellent and 7.1% good in Group B and 83.9% excellent and 16.1% good in Group L; patient satisfaction was 82.1% excellent and 17.9% good in Group B and 80.6% excellent and 19.4% good in Group L (*P* = 0.273 and *P* = 0.883 for surgeon and patient satisfaction, resp.).

Anesthesia side effects were infrequent and minor (Table 5), and symptoms were resolved completely during the first 24 h. Follow-up on days 1, 3, and 7 after surgery revealed neither neuroophthalmological symptoms nor other side effects.

## 4. Discussion

Levobupivacaine is proving increasingly popular to replace bupivacaine given its similar efficacy and fewer cardiovascular and CNS side effects. Its pharmacokinetic properties are similar to those of racemic bupivacaine. Several studies indicate that its faster protein binding rate suggests a lower degree of toxicity [6].

The majority of the clinical studies that have compared levobupivacaine and bupivacaine have discovered few differences between them and report that both anesthetics perform similarly. In their randomized, double-blind prospective study, Glaser et al. [7] compared isobaric solutions (3.5 mL of 0.5% levobupivacaine; 3.5 mL of 0.5% bupivacaine) in 80 patients undergoing elective hip replacements. These authors found no clinical differences and concluded that both drugs were equipotent and offered similar durations, onset times, and degrees of motor and sensory blockades. After comparing 3 mL of 0.5% spinal bupivacaine and levobupivacaine for hip surgery, Fattorini et al. [8] found that there were no significant differences in spinal blockade characteristics. Sathitkarnmanee et al. [12] conducted a study with 70 patients to compare 0.5% isobaric levobupivacaine (3 mL) versus 0.5% isobaric bupivacaine (3 mL) for elective lower limb and lower abdominal surgery with spinal anesthesia. These authors showed no significant differences in the quality of motor and sensory blockades between both groups. Lee et al. [9] undertook a study which included 50 patients awaiting urological surgery under spinal anesthesia. These authors employed 2.6 mL of 0.5% isobaric solution of levobupivacaine and bupivacaine and reported no significant differences. However, it did not include data on the time of two segment regressions and motor blockade duration. Vanna et al. [10] compared 0.5% hyperbaric bupivacaine and 0.5% isobaric levobupivacaine, 2.5 mL for both, for elective transurethral endoscopic surgery. They showed equally effective potencies for spinal anesthesia in both sensory blockade onset time and duration terms. Cuvas et al. [11] included these measurements and used the same doses as we have done in our study but used isobaric levobupivacaine and hyperbaric bupivacaine. They showed equal potencies for spinal anesthesia as far as sensory blockade duration and onset time are concerned. Yet levobupivacaine generally achieved more sustained sensory and motor blockades. The hemodynamic changes and adverse events in both were similar. In their randomized, double-blind, cross-over study with 80 healthy volunteers, Alley et al. [13] compared 0.25% hyperbaric bupivacaine and levobupivacaine for spinal anesthesia (4–12 mg doses). Both local anesthetics offered equivalent efficacy in sensory and motor blockades terms.

Our study demonstrates that 0.5% isobaric levobupivacaine and 0.5% isobaric bupivacaine are equally effective as spinal anesthesia in knee arthroscopy, which requires a sensory blockade of at least T12.

No statistically significant differences were recorded for the sensory blockade onset rate and extent between both local anesthetics for the onset time to T8, T4, time to maximum spread, and motor and sensory blockade duration. However, bupivacaine presented a faster onset time to T12, whereas the mean maximum sensory blockade level was higher for bupivacaine than for levobupivacaine.

Group B also obtained a quicker motor blockade onset and time to maximum motor blockade. Motor blockade onset is an important quality of the local anesthetic agent utilized for spinal anesthesia because a delayed onset time delays surgery from starting, which may prove time-consuming.

In the present study, bupivacaine density was more hypobaric than that of levobupivacaine. The local anesthetic injection rate, needle size, and patient position were kept constant. The higher maximum sensory block level obtained for bupivacaine may thus be related to its baricity.

Despite some studies providing evidence that levobupivacaine is less cardiotoxic and neurotoxic than bupivacaine [1–6], we found no differences between both agents for hemodynamics and incidence of side effects.

No neuroophthalmological effects were detected in either group. These results can be justified by insufficient local anesthetic reaching the cerebrospinal system; limited spinal fluid leak; type of surgery that entails no risk of ischemia or embolism. Further and larger studies are needed to assess postspinal anesthesia neuroophthalmological effects.

A similar interval between spinal injection and first voiding in both groups occurred. Group L required postoperative supplemental analgesia before Group B.

In conclusion, isobaric bupivacaine produces spinal blockade with a faster time to maximum motor blockade and to the onset of sensory and motor blockades than levobupivacaine, as well as a higher mean maximum sensory blockade level. Both local anesthetics offer equal efficacy in terms of motor and sensory blockade duration. The two study groups present similar hemodynamics and side effects. According to these data, levobupivacaine is a suitable alternative to bupivacaine for spinal anesthesia in knee arthroscopy because it is a well-tolerated anesthesia that offers similar effectiveness. Nonetheless, bupivacaine is more recommendable for not only surgery that requires greater sensory blockade and longer postoperative analgesia, but also for emergency operations where a delay in starting surgery cannot be permitted.

## Figures and Tables

**Table 1:** The neuroophthalmological symptoms questionnaire.

Symptoms	No	Yes
Headache		
Nausea		
Hypotension		
Shivering		
Dizziness		
Eye pain		
Blurred vision		
Dimness of vision		
Visual field loss		
Feeling of palpebral heaviness		
Photophobia		
Difficulty reading or focusing on close objects		
Diplopia		

**Table 2:** Patient characteristics and duration of surgery.

	Bupivacaine (*n* = 28)	Levobupivacaine (*n* = 31)	*P* value
Age (yr)	41 (14)	39 (14)	0.54
Height (cm)	169 (9)	173 (7)	0.06
Weight (kg)	77 (14)	77 (13)	0.99
Gender (M/F ratio)	19/9	26/5	0.15
ASA (I/II ratio)	18/10	26/5	0.08
Duration of surgery (min)	26 (16)	21 (10)	0.17

yr: year; cm: centimetres; kg: kilograms; M: male; F: female; min: minutes.

Data are expressed as means (SD). There were no significant differences.

**Table 3:** Characteristics of intrathecal blockades for bupivacaine and levobupivacaine.

	Bupivacaine (*n* = 28)	Levobupivacaine (*n* = 31)	*P* value
Time to sensory blockade onset (T12) (min)	1.5 (1–10)	3 (1-2)	0.018*
Onset to T8 (min)	5.5 (2–42)	9 (1–25)	0.495
Onset to T4 (min)	4.5 (3–26)	6 (4–53)	0.351
Highest sensory blockade level (dermatome)	T3 (T2–T12)	T6 (T2–T12)	0.008*
Time to maximum sensory blockade (min)	14 (5–42)	15 (2–53)	0.079
Sensory regression (min)	153 (20–312)	154 (52–317)	0.429
Motor onset time (min)	3 (1–15)	7 (1–15)	0.003*
Maximum motor blockade (patients with Bromage 2/patients with Bromage 3)	1/27	5/26	0.122
Time to maximum motor blockade (min)	15 (3–38)	40 (6–83)	0.014*
Motor block regression (min)	208.5 (130–357)	198 (55–310)	0.24
Time to micturate (min)	256.5 (95)	248 (90.64)	0.728
Time to analgesia (min)	297 (146–444)	247 (184–436)	0.025*

min: minutes.

*A significant result takes a *P* value of < 0.05. Data are presented as medians (range) or means (SD).

**Table 4:** Near best-corrected visual acuities values in both eyes.

	Median (range)			
	Right eye		Left eye	
	BS	AS	BS	AS
Levobupivacaine	1 (0.8–1)	1 (0.8–1)	1 (0.66–1)	1 (0.66–1)
Bupivacaine	1 (0.8–1)	1 (0.8–1)	1 (0.66–1)	1 (0.66–1)

BS: before spinal anesthesia; AS: after spinal anesthesia.

**Table 5:** Frequency of adverse events.

Adverse events, number (%)	Bupivacaine (*n* = 28)	Levobupivacaine (*n* = 31)
Hypoxia	0	0
Bradycardia	1 (3.6)	2 (6.4)
Hypotension	1 (3.6)	2 (6.4)
Headache	0	0
Nausea/vomiting	0	1 (3.2)
Shivering	1 (3.6)	1 (3.2)
